# Multicentre Study Into the Use of Polyethylene Glycol With Electrolytes Over at Least 6 Months to Treat Constipation in Paediatric Populations

**DOI:** 10.1097/PG9.0000000000000353

**Published:** 2023-10-05

**Authors:** Adolfo Bautista-Casasnovas, Federico Argüelles-Martín, Benjamín Martín-Martínez, María Jose Domínguez-Otero, Marta Tavares, Jorge Amil-Dias

**Affiliations:** From the * Pediatric Surgery Department, Hospital Clínico Universitario de Santiago de Compostela; † Pediatric Gastroenterology Department, Hospital Universitario Virgen Macarena, Seville; ‡ Pediatric Gastroenterology Unit, Hospital de Tarrasa, Barcelona; § Pediatric Department, Policlínico Vigo, Povisa, Vigo; ∥ Pediatric Gastroenterology Unit, Centro Infantil do Norte, Oporto; ¶ Pediatric Gastroenterology Department, Hospital Sao Joao, Oporto.

**Keywords:** bowel movement, childhood, functional constipation, long-term use, Macrogol 3350 with electrolytes

## Abstract

**Background:**

Constipation is a common clinical problem in children, for which the first-line therapeutic options are osmotic laxatives, mainly polyethylene glycol (PEG). These treatments are often prescribed for short or limited periods, with progressive treatment withdrawal often resulting in relapses. However, there are a few studies into the long-term use (≥6 months) of PEG 3350 with electrolytes (PEG+E) in terms of the patients’ clinical evolution.

**Objectives:**

To assess bowel movement and other relevant symptoms in children with constipation receiving PEG+E (≥6 months), as well as parent/caregiver satisfaction with this treatment.

**Methods:**

A retrospective, observational, descriptive, longitudinal, and multicentre study was carried out on 74 children diagnosed with functional constipation (ROME IV criteria) who had received PEG+E (≥6 months). Bowel control was assessed using the Bristol stool scale, and the parent’s/caregiver’s perception of the treatment was also evaluated employing a nonvalidated questionnaire.

**Results:**

Children with an average duration of constipation >1 year experienced a significant improvement in bowel movements and stool consistency when using PEG+E. The mean duration of use was 18.6 (±13.4) months, without the need to adjust the dose for weight. All clinical symptoms improved significantly except bloating, and all the parents/caregivers confirmed these clinical improvements.

**Conclusions:**

Children treated with PEG+E (≥6 months) normalised their bowel movements, improving the clinical symptoms related to constipation in the absence of serious advert events or the need for dosage adjustments due to weight gain. Parents/caregivers reported good satisfaction with PEG+E treatment.

**WHAT IS KNOWN**

- Constipation becomes chronic in a third of the paediatric patients.
- PEG 3350 with electrolytes (PEG+E) is the most widely used osmotic laxative in Europe, and it is normally prescribed for short or limited periods in children, such that there is little information regarding its long-term use (≥6 months).

**WHAT IS NEW**

- Symptoms of constipation were resolved in a cohort of 74 children with functional constipation (ROMA IV criteria) who received PEG+E for ≥6 months, with no reports of serious adverse events.
- Parents/caregivers reported satisfaction with PEG+E treatment.

## INTRODUCTION

Constipation is a common clinical problem with a variable prevalence in the paediatric population, depending in particular on the diagnostic criteria used (1,2). In children, one-third of cases become chronic (3), with premature withdrawal of treatment being the commonest cause of recurrence (4,5), and the symptoms of constipation often continue into adulthood (1,2). Given this high prevalence, constipation is the cause of many paediatric consultations and hospital admissions, and the extended use of laxatives contributes to the economic burden associated with this condition (1,2).

The most common treatment for constipation involves osmotic laxative use (1,2,6), mainly based on polyethylene glycol (PEG), with a widely reported effectivity (7,8). PEG 3350 with electrolytes (PEG+E) is currently the most widely used osmotic laxative in Europe (9), a high molecular weight iso-osmotic agent that increases the water content of the stool and improves colon motility of the softened stools and the mechanics of defection (7,9,10). Several randomised and controlled trials (9,11–14) or observational studies (10,15–19) have confirmed the efficacy and safety of PEG+E to treat faecal impaction and chronic constipation in the paediatric population (even in infants below 2 years of age), even following treatments of 6 months or less. However, to the best of our knowledge, only one randomised and controlled trial has assessed PEG+E use in children for more than 6 months (20) and thus, there is a clear need to further assess the performance of PEG+E-based regimes for longer-term treatment (≥6 months) of paediatric constipation.

Accordingly, we assessed the symptoms of children with functional constipation treated with PEG+E for at least 6 months, evaluating the number of bowel movements per week and the improvement in stool consistency according to the Bristol stool scale as the primary endpoints (21). The secondary endpoints involved the description of the epidemiological and clinical characteristics of the children included at baseline, the evaluation of the PEG+E doses used (faecal disimpaction dose, treatment dose, and maintenance dose), the evolution of the symptoms of constipation, and finally, the subjective satisfaction of the parents or caregivers with the treatment.

## PATIENTS AND METHODS

### Study Design

This is a retrospective, observational, descriptive, and longitudinal study in which paediatric patients were recruited from gastroenterology clinics at 5 different tertiary hospitals. Children from 2 to 15 years of age at diagnosis with functional constipation according to ROME IV criteria (22) and receiving PEG+E as a treatment for at least 6 months were included in the study. Exclusion criteria included the use of other laxatives concomitantly with PEG+E; having abandoned PEG+E treatment for a period greater than 30 days; having an organic pathology related to constipation (anal atresia, Hirschsprung disease, etc.) and lack of retrospective data referring to visit T0.

### Methodology

T0 was defined as the time at which PEG+E treatment (Movicol Paediatrics, Norgine BV, Amsterdam, Netherlands) was prescribed, at which point the baseline characteristics of the patient’s constipation were defined. T1 is the point of inclusion and cut-off time of the study, at which point the constipation status of the patients was also defined.

Potentially eligible patients completed the ROME questionnaire when they were invited to participate in the study, and their parents/caregivers were informed and approved their participation. A clinical consultation was performed at time T1 in which the participants were interviewed and the paediatric gastroenterologist assessed the children’s constipation status based on the ROME IV criteria (22).

Epidemiological and clinical information were collected through a retrospective review of the patient’s medical history and through interviews: the age; weight and height at T0 and T1; gender; advert events (AEs); personal and family history; PEG+E doses (g/kg of weight) as a faecal disimpaction dose, treatment dose and maintenance dose, and duration of constipation treatment. Reports on the progress of bowel movements per week were compiled, as well as data from the Bristol stool scale (1–7) (21). The child’s symptoms (abdominal pain, bleeding, fissures, presence of fecalomas, soiling, bloating, or incontinence) were assessed at baseline (T0) and at T1, and if any information was not registered in the patient’s medical history, this was collected at the interview.

The parent/caregiver satisfaction with the treatment was measured using a 5-point Likert scale (1 = totally disagree; 2 = disagree; 3 = indifferent; 4 = agree; and 5 = totally agree), employing a nonvalidated questionnaire consisting of 4 statements: (S1) PEG+E was an effective treatment for constipation (efficacy of PEG+E); (S2) The child’s symptoms of constipation responded favourably to PEG+E administration (symptom improvement); (S3) The treatment was easy to take (adherence); and (S4) Overall, the treatment was satisfactory.

The study was approved by the Regional Clinical Research Ethics Committee on December 16, 2015 (Registration Code: 2015/715), and by the Spanish Agency for Medicine and Health Products (AEMPS: identification number ABC-PEG-2015-12).

### Statistical analysis

A descriptive univariate and bivariate analysis was carried out using the IBM SPSS software package (SPSS). Qualitative variables were described by their frequency and percentage, and quantitative variables as their mean and standard deviation (SD), all of which followed a normal distribution. The differences between the mean values at T0 and T1 were determined with a Student *t*-test for independent samples. For all comparisons, a *P* value of ≤0.05 was considered statistically significant.

## RESULTS

A total of 74 children with functional constipation between 2 and 12 years of age were recruited for this study, 55.4% male and 44.6% female. The mean (±SD) duration of the symptoms of constipation before starting PEG+E treatment was >1 year (15.6 ± 8.4 months) and more than half of the patients (66.2%) had a family history of constipation, although only 32.43% (n = 24) had been previously treated with other laxatives. Faecal disimpaction was necessary in 49 children (66.2%) and the mean PEG+E dose used was 1.0 (±0.8) g/kg. By contrast, the mean treatment dose at T0 was 0.45 (±0.25) and 0.34 (±0.23) at T1 (Table 1).

**TABLE 1.** Age, weight, and polyethylene glycol with electrolyte solution (PEG+E) dose at visits T0 and T1

	Visit T0	Visit T1
Age (years), mean (SD)	5.7 (±3.6)	7.6 (±3.8)
Weight (kg), mean (SD)	23.9 (±10.4)	28.5 (±14.2)
PEG+E (g/Kg of weight), mean (SD)	0.45 (±0.25)	0.34 (±0.23)

**FIGURE 1.**
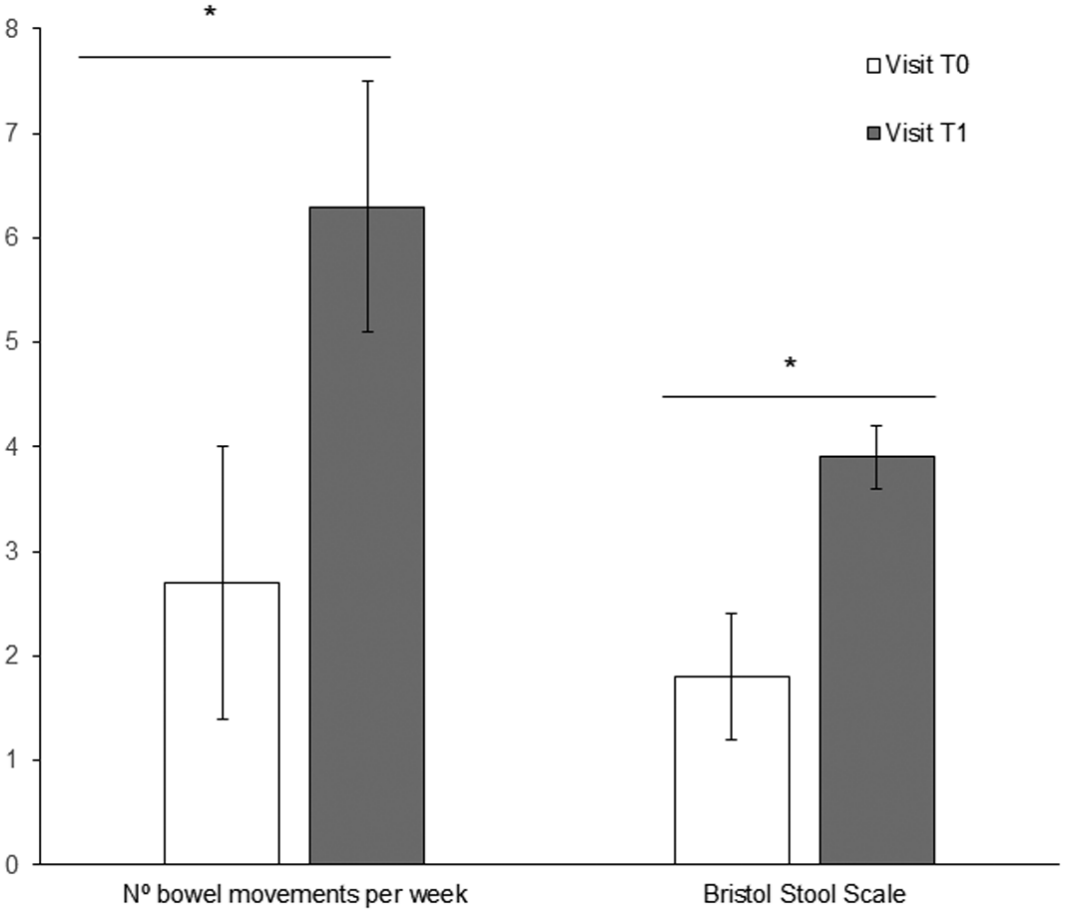
Bar chart showing the mean (±SD) bowel movements per week and the mean (± SD) Bristol stool scale values at T0 and T1. Both parameters had significantly higher means at T1 than at T0 (*P < 0.05, Student *t*-test).

The mean duration of PEG+E use was 18.6 (±13.4) months (range 8–73 months), and 59.45% (n = 44) of the patients took the treatment for more than 1 year. The follow-up data showed that the number of bowel movements per week increased to more than 4 after 2.8 (±1.5) weeks of treatment, and 81% (n = 60) of the patients achieved 4 or more weekly bowel movements after having taken PEG+E for at least 3 weeks. We found that children recorded significantly more weekly bowel movements and achieved a better Bristol scale score at T1 relative to T0 (*P* < 0.05 for both parameters, Student’s *t*-test: Fig. 1).

**FIGURE 2.**
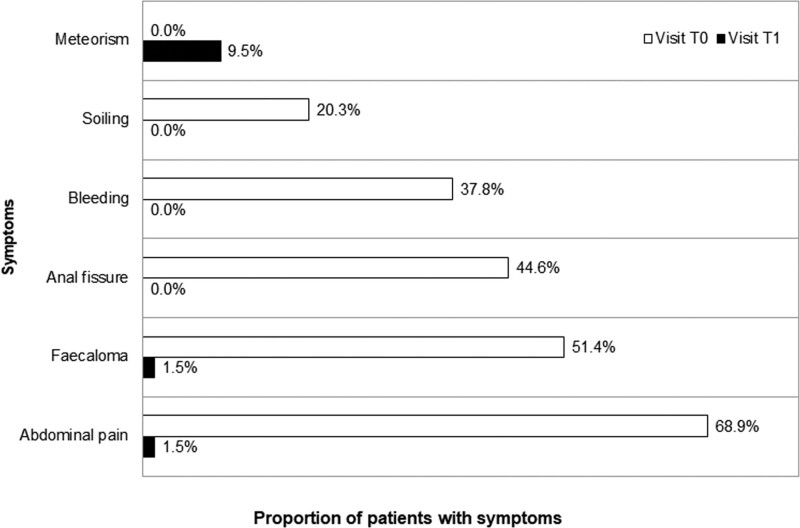
Bar chart showing the proportion of children who suffered each symptom at T0 or T1.

The proportion of patients with clinical symptoms was recorded at T0 and T1 (Fig. 2), and the most frequent symptoms suffered by the children at baseline were abdominal pain (68.9%), fecaloma (51.4), anal fissure (44.6%), bleeding (37.8%), and soiling (20.3%). All clinical symptoms were reduced considerably, with the resolution of the anal fissures, bleeding, and soiling in all patients. Bloating was a symptom that was attributed to 7 patients (9.5% of the total), in 1 case associated with encopresis.

**FIGURE 3.**
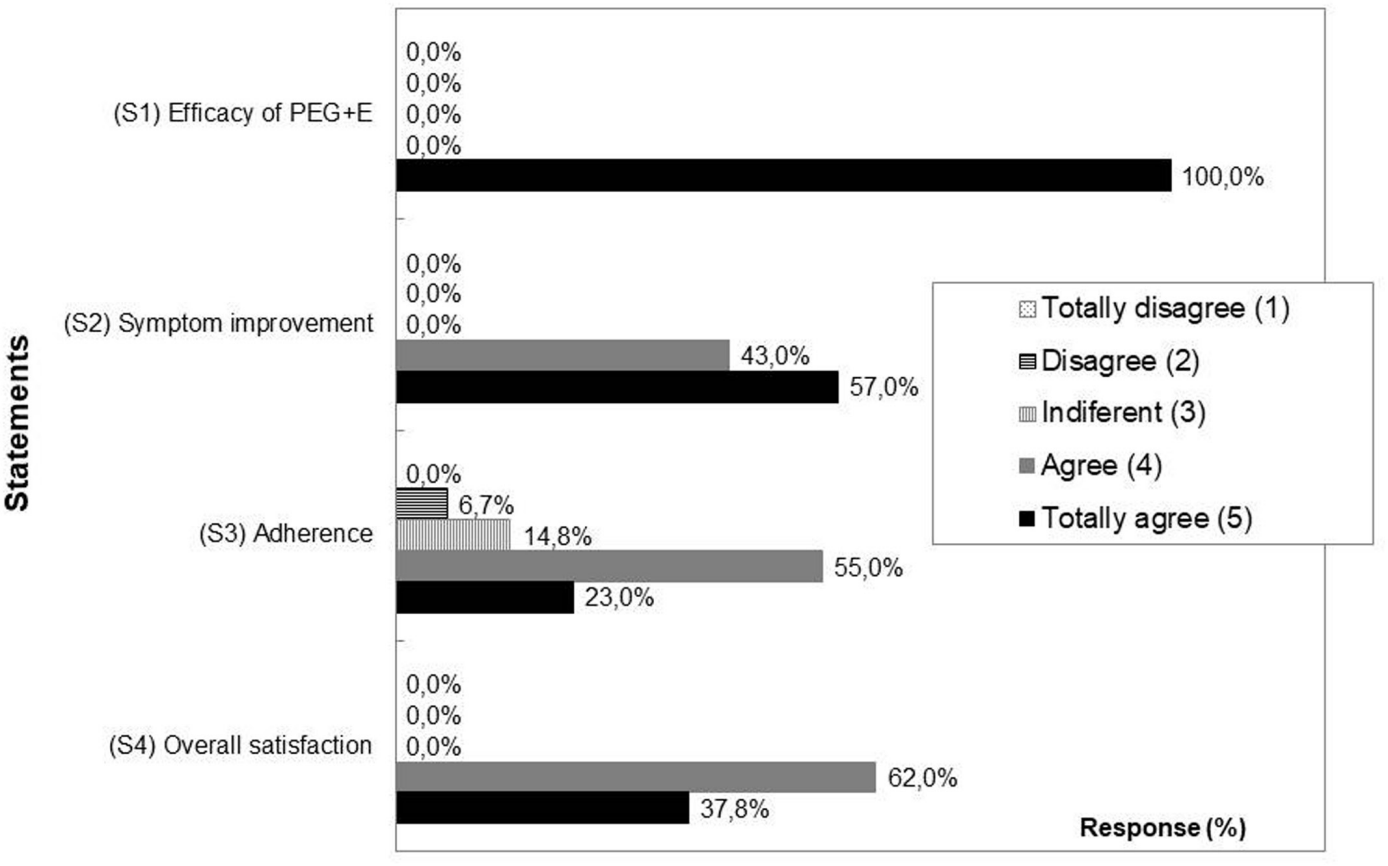
Bar chart showing the relative responses of the parents or caregivers to the satisfaction questionnaire on a 5-point Likert scale (1 = totally disagree; 2 = disagree; 3 = indifferent; 4 = agree; 5 = totally agree). The statements were: (S1) PEG 3350 with electrolytes (PEG+E) was an effective treatment for constipation (efficacy of PEG+E); (S2) The child’s symptoms of constipation responded favourably to PEG+E administration (symptom improvement); (S3) The treatment was easy to take (adherence); and (S4) Overall, the treatment was satisfactory.

All the parents or caregivers interviewed (100%) confirmed the efficacy of the PEG+E treatment (Fig. 3), with virtually 100% of them confirming (agree or totally agree) the improvement in the symptoms and their overall satisfaction with the treatment. Finally, around 80% of the parents/caregivers agreed or totally agreed with the ease of adherence to the treatment (Fig. 3).

## DISCUSSION

Despite the common use of osmotic laxatives to manage constipation in children, there is still a lack of clear evidence to support this practise. In recent years, the widespread introduction of PEG in paediatric practise has led to a resurgence in research on paediatric constipation (8). PEG is recommended as a first-line laxative for both faecal disimpaction and maintenance treatment for functional paediatric constipation (20,23,24). However, caregivers may be hesitant to administer medication over long periods due to a fear of a rebound effect or addiction (23). Indeed, early withdrawal of laxatives is the commonest cause of recurrence (4,5), highlighting the need for longer follow-up studies (8). Here, this need was addressed by assessing the long-term use of PEG+E (≥6 months) and its effects on the evolution of symptoms in paediatric patients with prolonged constipation (20,25–28). The results offer reassurance regarding the use of PEG+E in children with functional constipation over 8–73 months, improving bowel movements per week and stool consistency without provoking any serious AEs. The treatment also improved other symptoms, such as abdominal pain, fecaloma, anal fissure, bleeding, or soiling.

A positive family history of functional constipation is considered a risk factor for paediatric constipation (29,30), which has been associated with genetic factors (31) and with specific clinical characteristics, such as an earlier age at onset and a longer duration of the symptoms of constipation (32). Indeed, 66.2% of the paediatric population studied here had these characteristics, slightly higher than the proportions described elsewhere (ranging from 28 to 55%) (33–35). Only 32.43% of the population studied had received treatment for constipation before PEG+E administration, despite suffering symptoms of constipation for over a year. Nevertheless, this characteristic did not prevent them from achieving recovery in our study, consistent with existing evidence that there is no association between positive family history and recovery from constipation (36). This apparent contradiction between prolonged symptomatology and the absence of treatment could be explained in some degree by the environment of constipated families, whereby constipation symptoms may be underestimated and visits to a doctor postponed, therefore delaying treatment. In this regard, education has been considered to be equally important as medical therapy for this condition, and this should include counselling families to recognise the consequences of withholding behaviours, and the benefits of behavioural interventions, as well as an expectation of a chronic course with prolonged therapy, frequent relapses, and a need for close follow-up (8,22,24).

This study did not evaluate the quality of life of the constipated children but given the prolonged treatment duration, we assume that families and children suffer a worse quality of life, as observed previously (37). Also, the economic impact on the family of long-term laxative use was not evaluated, although it would be reasonable to assume some impact in this regard. Although separating costs attributed to laxative use from those due to other medical conditions in children with constipation may be difficult (38), our subjective impression was that, in some cases, early withdrawal from treatment may be due to the cost of treatment, which would justify further studies designed to address such a specific question (39) and to analyse the long-term acceptance of this treatment.

Regarding parent/caregiver satisfaction, although its assessment was totally subjective (through a nonvalidated scale), and therefore its results must be interpreted with caution, the high perception of efficacy (100%) and overall satisfaction (100%) with the treatment appear to be remarkable. Another important issue is related to adherence, as children may not like the taste of PEG+E^23^. However, 78% of the caregivers confirmed the ease of adherence to the treatment, a positive indication of the willingness of caregivers to administer the PEG+E regimen, which is a key element in the acceptance of any paediatric treatment (40).

Although the efficacy of PEG is dose-dependent, there is no established dose for its use (19,41), such that the effective doses for faecal disimpaction and maintenance differ between studies (8,17,22). Here, the faecal disimpaction dose (in g/kg of weight) was approximately twice the dose at visit T0, with a slightly lower dose at visit T1 than at T0. This decrease was mainly due to the children’s weight gain and the absence of any need for dose adjustment. Similar outcomes have been described in other paediatric populations receiving long-term PEG regimens (≥6 months) (42–44). Together, these results could indicate that these regimens do not induce tolerance and that they might even facilitate some type of bowel retraining.

This study has important limitations, the most relevant of which is its retrospective nature, such that certain AEs (especially non-serious AEs), the administration of rescue medication, or discrepancies between the medication prescribed and that actually used may not be included in the medical records. To minimise this reporting bias, data collection was completed with a retrospective study of the case histories. Another limitation is the lack of a placebo arm to properly evaluate the efficacy of PEG+E treatment, although its efficacy in the constipated paediatric population has been demonstrated previously in randomised and controlled trials (9,11–14,20). Finally, the description of symptoms depended on the parent or caregiver, as did the assessment of treatment satisfaction, potentially introducing a reporting bias.

In conclusion, despite the limitations that this is an observational, retrospective, and single-arm study carried out on only 74 children, our results suggest that the use of PEG+E in children with constipation for ≥6 months enhances bowel movements, normalises stool consistency, and reduces other symptoms in the absence of serious AEs. Moreover, parents/caregivers reported a high level of satisfaction with the treatment, even in the case of children with a long-standing history of functional constipation. Finally, PEG+E was used for more than a year and a half without the need to adjust the dose for weight, decreasing the doses used in g/kg of weight while maintaining the improvements in constipation.
